# Mental Health First Aid training and assessment in Australian medical, nursing and pharmacy curricula: a national perspective using content analysis

**DOI:** 10.1186/s12909-022-03131-1

**Published:** 2022-01-29

**Authors:** Lily Pham, Rebekah Jane Moles, Claire Louise O’Reilly, Mary Joy Carrillo, Sarira El-Den

**Affiliations:** 1 Sydney Pharmacy School, Faculty of Medicine and Health, The University of Sydney, A15 Pharmacy and Bank Building, Science Rd, Camperdown, NSW 2006 Australia; 2 School of the Arts and Media, Faculty of Arts and Social Sciences, The University of New South Wales, Robert Webster Building G14 Room 312, Kensington, NSW 2052 Australia

**Keywords:** Mental health first aid, Education, Pharmacy, Medicine, Nursing, Curricula

## Abstract

**Background:**

Suicide is among the leading causes of death among people aged 15 to 29 worldwide. Healthcare professionals interact with people at risk of suicide regularly, yet mental health and crisis first aid training is lacking in curricula. Mental Health First Aid (MHFA) training teaches crucial communication and crisis first aid skills and is increasingly recognised as integral to healthcare education. This study aimed to explore the extent of, as well as barriers and enablers to MHFA training delivery and assessment in Australian medical, nursing and pharmacy curricula.

**Methods:**

All accredited Australian medical, nursing and pharmacy program providers were identified through Australian Health Practitioner Regulation Agency and National Boards websites and invited to participate in a semi-structured interview. A purpose-designed interview guide explored if and how MHFA training was delivered and assessed in curricula, as well as perceptions of and barriers and enablers to MHFA training. Interview recordings were transcribed verbatim, allowing for deductive content analysis to compare MHFA training provision across programs.

**Results:**

Of 75 invited program providers, 36 (48%; 13 medical, 13 nursing and 10 pharmacy) participated, of which 15 representatives (42%; six medical, two nursing and six pharmacy) reported providing MHFA training to students. Differences in mandating training, year level of students completing training, type of training delivered and source of MHFA instructors were identified. Barriers to MHFA implementation included perceived adequacy of existing curricula, lack of funding and time, while facilitators included perceived benefit and availability of funding.

**Conclusion:**

MHFA training is provided to more than one third of medical, nursing and pharmacy students in Australia. Increased funding may facilitate the integration of MHFA as a minimum standard of mental health training for future healthcare professionals. Further research exploring the effectiveness of MHFA in improving behaviours and its impact on patient outcomes is warranted.

**Trial registration:**

This study was approved by the University of Sydney Human Research Ethics Committee [Project number: 2020/087].

## Background

Suicide is the fourth leading cause of death globally among young people aged 15 to 29 [1] and yet many healthcare professionals who interact with people at risk of suicide do not have adequate mental health and suicide prevention training, skills or confidence to intervene [2]. Mental Health First Aid (MHFA) is an accredited training program designed to help people identify and support others experiencing mental health problems and crises [3]. MHFA training is now available in over 25 countries internationally and is built on the principles of physical first aid [4]. Training also includes a MHFA Action Plan named ALGEE, prompting participants to *“approach the person, assess and assist with any crisis; listen and communicate non-judgementally; give support and information; encourage the person to get appropriate professional help and encourage other supports”* [5]. MHFA is however, not designed to substitute pre-existing mental health education taught in curricula but rather, benefits students in gaining knowledge, improving attitudes and skills in managing mental health crises [6].

Although approximately 1% of the adult Australian population has completed MHFA training [7], the extent of training among healthcare students and professionals is unknown and healthcare curricula continue to lack adequate training on suicide prevention [8]. An independent review of Australian nursing curricula highlighted insufficient mental health expertise to accommodate for the growing demand for mental healthcare, noting that current accreditation standards allow for inconsistencies in mental health education delivery as higher degree providers are able to choose what content to include [9]. This lack of mental health crisis training and consistency in core healthcare curricula is also echoed in psychiatry [10] and pharmacy [11] programs where the disparity between burden of mental illness and time allocated to mental health teaching is apparent [10]. Standardised implementation of MHFA in medical, nursing and pharmacy curricula may address such disparity in providing a minimum level of crisis care training.

Natural disasters, including the Australian bushfires [12], and COVID-19 pandemic have contributed to increased risk factors associated with mental health decline [13], placing unprecedented stress on the healthcare system and exemplifying the need for increased mental health support. MHFA training for healthcare professionals may allow for appropriate assessment and support of people experiencing mental health problems and crises [14, 15]. MHFA training has demonstrated its value in significantly improving confidence, mental health literacy and stigma in medicine [16], nursing [17] and pharmacy [18] students. With doctors, nurses and pharmacists having higher suicide rates than most other professions [19] and representing over 75% of the healthcare workforce [20], training is needed to not only support patients they regularly interact with, but also frontline healthcare students and workers, themselves.

In 2012, Australian Government funding was provided for first-year health, allied health and human services tertiary students to complete Tertiary-MHFA training. However, funding was prematurely exhausted due to high demand [21]. In May 2020, Medical Deans Australia and New Zealand (MDANZ) were granted Australian Government funding for 2 years of online MHFA training [22]. Similarly, funding was granted for pharmacists and nurses working in bushfire- [23, 24] and drought-affected [25] communities, recognising pharmacists as first responders [26]. Hence, the cost of MHFA training needs to be considered prior to implementation and may be available through internal sources or government grants; however, funding can be scarce, short-term and in high demand.

Although current Australian legislation does not mandate MHFA training for healthcare professionals, MHFA can be mandated for professionals, such as frontline staff of the Northern Ireland Prison Service [27]. There are also an increasing number of workplaces in Australia that are now recognised as offering MHFA training to staff [28]. There is evidence to suggest that MHFA training is increasingly implemented in university curricula for not only students studying health-related programs, but also for life, physical and pharmaceutical sciences [15]. With increasing citations in the literature stating the need for MHFA as a pre-requisite in work places [29–31] and a need for increased mental health support, this study aimed to explore the extent of, as well as barriers and enablers to MHFA training delivery and assessment among Australian medical, pharmacy and nursing program providers.

## Methods

### Identification of participants

All accredited Australian medical, nursing and pharmacy program providers were identified using the Australian Health Practitioner Regulation Agency (AHPRA) and National Boards websites [32]. For the purpose of this manuscript, a “program provider” was defined as an accredited provider of a nursing, medical or pharmacy program that enables graduands to apply for registration as a nurse, doctor or pharmacy upon graduation. Publicly available information was used to determine relevant staff, such as Deans and Education Leads/Representatives to contact for participation, via email starting May 1st, 2020. Prospective participants were sent follow-up invitations up to three times until August 11th, 2020. Contacts were encouraged to refer more appropriate staff for participation, if needed. Consenting participants engaged in a semi-structured interview, via telephone, between May 8th and July 21st, 2020.

### Interview guide

A research team of three pharmacists with mental health research expertise, of which two are accredited MHFA instructors, developed the interview guide based on findings from a systematic review on MHFA training and assessment among university students [15]. The interview guide included open and closed-ended questions to investigate the process used and reasons for MHFA delivery. Where MHFA was delivered, the first section of the interview guide involved questions relating to whether training was compulsory, length of time MHFA was offered, student cohorts receiving training, type of training provided (standard, blended or eLearning), source of MHFA instructors, and assessments/activities related to MHFA skills, post-training. Where MHFA was not provided, the first section involved questions relating to whether MHFA training was being considered, and what, if any, other types of related training were delivered. The second section of the guide explored participants’ perceptions of the value, benefit, enablers and barriers relating to MHFA training delivery and assessment in healthcare education. This second section was only asked where MHFA was not provided by the relevant program(s). The interview guide was piloted with an experienced qualitative researcher external to the study prior to data collection.

### Data analysis

Interviews were audio-recorded and transcribed verbatim. Data was extracted, tabulated and comparatively analysed in-text. Data highlighted whether training was compulsory, length of time MHFA was offered, student cohorts receiving training, type of training provided, source of MHFA instructors, and assessments/activities related to MHFA skills post-training. Deductive content analysis was used to analyse transcribed interview data [33]. One author (LP) immersed herself in the data by re-reading transcripts and coding responses. Coding was shared with other authors during regular meetings until four authors (COR, LP, RM, SED) reached a consensus on data analysis.

#### Programs offering MHFA

A data extraction table (Table 1) was created to allow for comparative analysis through extraction of the following MHFA-specific data: compulsory or voluntary implementation, duration of years offered, courses run by provider per year, proportion of the student cohort completing MHFA and reason for proportion (if voluntary), type of MHFA training, source of instructors, exemptions available, student cohort assigned MHFA and post-training assessments related to MHFA. Type of MHFA training delivered was categorised as Standard, Blended or eLearning. Standard MHFA training is delivered in a 12-h face-to-face (FTF) format [34]. Blended MHFA training is delivered through self-directed eLearning modules followed by an instructor-led component (delivered FTF or online) [21]. When only eLearning is undertaken, this refers to an online, self-directed learning component of MHFA that alone, does not enable participants to become accredited to provide MHFA, better known as Mental Health First Aiders (MHFAiders) [21].

Data relating to other types of mental health-related training provided within programs or offered by their university more broadly (including MHFA) was not extracted. Data extraction was specific only to MHFA training provision within medical, nursing and pharmacy curricula only, to ensure relevance to the study aims. Nonetheless, this data, when relevant, was reported in-text.

#### Programs not offering MHFA

Responses by the representatives of program providers that did not offer MHFA training were deductively content analysed and reported separately in-text in relation to section 2 of the interview guide.

## Results

### Study recruitment and participation

Of the 77 program providers (22 medical, 37 nursing, 18 pharmacy) with accredited programs of study [32], 75 program providers (22 medical, 35 nursing and 18 pharmacy) were contacted for participation. Two non-university nursing program providers were not invited as their contact information was unavailable publicly. Of the 75 program providers contacted, 45 responded (56% response rate), of which representatives from 36 (13 medical, 13 nursing and 10 pharmacy) consented to be interviewed. At the time of this study, this represented 59, 35 and 56% of all medicine, nursing and pharmacy program providers in Australia. Of the nine program providers that declined participation, six representatives indicated that their program(s) did not offer MHFA training and three did not specify.

Program providers represented were geographically distributed across all Australian states and territories except for the Northern Territory (NT). Medical program representatives (*n* = 13) were interviewed across New South Wales (NSW) (*n* = 4), Queensland (QLD) (*n* = 4), Western Australia (WA) (*n* = 2), Victoria (VIC) (*n* = 1), South Australia (SA) (*n* = 1) and Tasmania (TAS) (*n* = 1). Nursing program representatives (*n* = 13) were interviewed across NSW (*n* = 6), QLD (*n* = 2), VIC (*n* = 1), ACT (*n* = 1), WA (*n* = 1), SA (*n* = 1) and TAS (*n* = 1). Pharmacy program representatives (*n* = 10) were interviewed across NSW (*n* = 4), WA (*n* = 2), QLD (*n* = 2), VIC (*n* = 1) and ACT (*n* = 1).

All interviewees were involved in mental health teaching, student well-being activities and/or had senior leadership positions (e.g., Head of Discipline). Fifteen program providers (seven medical, two nursing, six pharmacy) provided MHFA within their program(s), while 21 (six medical, 11 nursing, four pharmacy) did not at the time of interview. However, nine of these 21 program provider representatives (six medical, one nursing, two pharmacy) reported an intention to provide MHFA training in 2020 or 2021.

### Characteristics of MHFA training delivered by program providers (*N* = 15)

Table 1 presents the characteristics of MHFA training among 15 program providers who provided MHFA training within their program, at the time of interview.

**Table 1:** Characteristics of MHFA provision among program providers (*n* = 15)

			***If compulsory***	***If Voluntary***	***If Voluntary***						
***Code***	***Compulsory/ Voluntary***	***Duration of years MHFA offered***	***Courses run by provider per year (courses)***	***% of student cohort completing MHFA (%)***	***Reason for %***	***Mode of MHFA training delivery***	***Source of MHFA instructors***	***Exemptions to MHFA training***	***Student cohort completing MHFA training***	***Opportunity to practice?***	***Assessed?***
**Medicine**											
M1	Compulsory	3	1	N/A	N/A	Blended	Internal Staff	None provided	1st of 5-year program; undergraduate.	No	No
M2	Compulsory	4–5	1	N/A	N/A	eLearning only	External instructors	None provided	Prior to 1st of 4-year program; postgraduate.	No	No
M3	Compulsory	2	1	N/A	N/A	Blended	Internal Staff	Current MHFA accreditation	1st and 2nd of 6-year program; undergraduate.	Yes	No
M4	Voluntary	4	N/A	75	1st come 1st serve	Blended	Internal Staff	N/A	1st of 4-year program; undergraduate. Note: may offer to other years due to MDANZ funding.	No	No
M6	Compulsory	6	N/A	N/A	N/A	eLearning only	External instructors	Current MHFA accreditation	1st of 4-year program; postgraduate.	No	No
M8	Voluntary	2	N/A	100	Aims to have all students qualified	Blended	Internal Staff	N/A	4th or 5th of 5-year program; undergraduate.	No	No
M10	Compulsory	Compulsory: 1; Voluntary: > 3	1	N/A	N/A	Blended	External instructors	Current MHFA accreditation	1st of 5-year program; undergraduate.	No	No
**Nursing**											
N2	Voluntary	> 7	Ad hoc	20	Based on funding, availability of trainers	Standard or blended	Internal Staff	N/A	All students: undergraduate.	No	No
N11	Compulsory	~ 6	4–5	N/A	N/A	Blended	Internal Staff	Current MHFA accreditation	1st and 2nd of 3-year program; undergraduate.	No	No
**Pharmacy**											
P1	Compulsory	5	> 3	N/A	N/A	Standard	Internal Staff	Students majoring in international, industry pharmacy or honours	4th of 4-year program; undergraduate. 2nd of 2-year program; postgraduate.	Yes	Yes
P2	Compulsory	> 6	1	N/A	N/A	Previously standard, now blended	Internal Staff	Current MHFA accreditation	Previously 2nd of 2-year program, now 1st of 2 year program; postgraduate.	No	No
P3	Compulsory	Compulsory: 2nd; previously voluntary. MHFA offered for 4 years.	1	N/A	N/A	Standard	Internal Staff	None provided	4th of 4-year program; undergraduate. 2nd of 2-year program; postgraduate.	Yes	No
P7	Compulsory	3–4	Unknown^a^	N/A	–	Blended	External instructors	Current MHFA accreditation	4th of 4-year program; undergraduate.	No	No
P8	Voluntary	3	1	30	1st come 1st serve	Blended	Internal Staff	–	Internship (5th) year.	No	No
P9	Voluntary	4	–	60	1st come 1st serve	Standard	External instructors	–	All students but focus on 4th of 4-year program; undergraduate.	No	No

*Abbreviations*: *M* medical program provider, *N* nursing program provider, *P* pharmacy program provider, *MHFA* Mental Health First Aid, *hr* hour, *N/A* not applicable

^a^: interviewee could not recall this information

#### Reasons for providing MHFA training within programs

Ten (five medical, one nursing, four pharmacy) of 15 program provider representatives reported that MHFA training was a compulsory component of curricula, of which two (M2 and M6) only rendered the eLearning component compulsory. However, the M6 representative reported their University offered the instructor-led component to interested students whilst the M2 representative did not specify any provision of this component. The M1 and M10 representatives reported that students supported the integration of MHFA, in that *“it also had the backing of the student organisations,”* (M1). Other reasons for compulsory integration included ensuring attendance predictability and demonstrating the importance of training to students, with the M3 representative reporting, *“we wanted to quarantine time in our program for students to undertake the task… if we made it optional, we wouldn’t necessarily reach the students who we felt particularly would benefit from the MHFA skills… we wanted to highlight that we see these skills as really important”.* The N11 representative reported that MHFA training was compulsory for their students because it, *“had benefit to help students transition from a lay person’s level of mental health literacy”* even though *“it doesn’t meet the standard for clinical practice”.* Four pharmacy program representatives (P1, P2, P3, P7) reported that MHFA training was compulsory in their programs, as it was “*essential for pharmacists’ practice…”* (P3) and ensured students were *“work-ready” (*P3, P7). It was recognised that training was beneficial not only for supporting patients, but also as *“peer support”* (P3), and *“for their own personal self-management”* (P7) as healthcare professionals, themselves, by improving awareness and skills.

Five (two medical, one nursing, two pharmacy) of 15 program providers offered MHFA training on a voluntary basis, only, of which four representatives (M4, N2, P8, P9) stated lack of funding was a reason for voluntary implementation or barrier to compulsory delivery, or both. Although M8 program representative reported that there was an aim to have all students MHFA accredited, the decision for ‘*voluntary, but recommended’* MHFA training was due to *“understaffing with one trainer to 1000 students at any one time”*. Additionally, the N2 representative highlighted timetabling was also an issue, as “*it would be great to have MHFA made a compulsory component, but in nursing, the curriculum is crowded so it’s a matter of prioritising*”. Hence, lack of resources and infrastructure were barriers to providing MHFA training to all students – as reported by M6, some programs simply did not “*have the ability to run FTF”.* Furthermore, there were concerns that mandating the course would *“take some of the joy out of it (MHFA) for students,”* although MHFA accreditation was, *“more of a value, adding to their life,”* (M8).

#### Duration of implementation

MHFA training was established in Australia in 2000 and has since gained national and international recognition [3, 7, 35]. Duration of implementation in Australian medical, pharmacy and nursing curricula ranged from integration for two consecutive years (M3) to at least 7 years (N2) at the time of interview (Table 1).

#### Mode of training delivery

Medical program providers preferred blended delivery with five of seven program providers offering Blended MHFA training and two only offering the eLearning component. Of the two nursing program providers offering MHFA, N2 offered the course to students who were successful applicants of a scholarship, covering Standard or Blended MHFA training costs, while N11 offered Blended MHFA exclusively. Of the six pharmacy program providers offering MHFA, three (P1, P3, P9) delivered Standard and three (P2, P7, P8) delivered Blended training. Program providers P2 and P8 both offered Blended MHFA training at the time of the interview, but had delivered Standard MHFA training previously, with P2 reporting that the blended format was more time-efficient, requiring less instructor-led course time.

#### Source of accredited MHFA instructors

Ten of 15 program provider representatives (four medical, two nursing, four pharmacy) reported that internal staff members with MHFA instructor accreditation delivered the training, while five (three medical, two pharmacy) employed external instructors.

#### Student cohorts receiving training

Four of seven medical program providers (M1, M4, M6, M10) delivered MHFA training to students in the first year of the degree, one (M2) prior to commencement of first year, one (M3) over the first and second year and one (M8) in the fourth or fifth year. Among nursing program providers, N2 offered MHFA training to students across all years and N11 offered MHFA training over the first and second year. MHFA training was offered in the final year for four (P1, P3, P7, P9) of six pharmacy program providers. This could have been due to practical reasons, including embedding MHFA into a unit of study where, “*it aligned [with] a lot of professional practice skills [taught] in that unit of study”* (P1). One pharmacy program provider (P2) that offered MHFA training in the first year had previously delivered the program in the final year. No reason was specified for this change. Another pharmacy program provider (P5) offered MHFA training to graduates participating in an intern training program as part of their postgraduate, pre-registration year.

#### Post-training activities and assessments

Only three program providers (M3, P1, P3) facilitated opportunities to practice MHFA skills post-training. Activities included scenario-based role plays of mental health crises, such as suicidal thoughts and behaviours. Program provider representatives reported using paid actors (M3) or people with lived experience of mental illness (P1) to participate in simulated role-plays. The P3 representative did not specify using paid actors or people with lived mental health experience when simulating patient scenarios. The P1 representative highlighted that post-training simulated (role-play) competency-based assessments made students, *“more competent in their skills and being able to provide support to people in a mental health crisis”*.

Twelve program providers (six medicine, two nursing, four pharmacy) did not provide formal opportunities for students to practice MHFA skills post-training. However, nine (five medicine, one nursing, three pharmacy) representatives noted that opportunities may arise coincidentally, for example when on clinical placement or *“as part of their mental health and psychiatry rotation,”* (M6). The P8 representative reported that there were informal opportunities to practice MHFA, “*in the course and more so in their jobs”*. One representative acknowledged the time constraints in providing opportunities to practice since they *“do that course towards the end of their degree… there’s not a lot of time,”* (P7). The M4 program provider offering voluntary MHFA felt that they could not assess students on a non-compulsory component of the course. Many representatives reported not providing post-training assessments outside of the assessment required for MHFA accreditation, with one representative explaining that, *“I don’t retest them on the MHFA skills because… that’s not part of our core curriculum,”* (N11). Another representative from N2 explained that while they did not have MHFA-specific assessments, *“what we teach in mental health subjects is about mental health so there’ll be overlap,”* .

### Program providers that did not provide MHFA training (*N* = 21)

Six medical, eleven nursing and four pharmacy program providers did not provide MHFA at the time of interview. All six medical program providers intended on providing the MHFA eLearning due availability of MDANZ funding: “we are offering … students access to the MHFA online learning packages on the back of a deal procured… by the MDANZ”, (M5). However, completion of the funded eLearning would not result in accreditation as a MHFAider. Representatives from three (M9, M11, M12) of these six medical programs stated that students seeking formal MHFA accreditation would be required to self-fund the instructor-led component required for accreditation. Two medical program providers representatives (M7, M13) reported considering arranging the delivery of the instructor-led component for their students. One medical program provider (M5) did not mention any instructor-led component delivery. Although formal MHFA training was not provided, four of six medical program provider (M5, M9, M11, M13) representatives reported that content related to suicide prevention, mental health and/or crisis management was embedded in their programs. Two medical program providers (M7, M12) did not specify whether additional mental health training was provided.

Although eleven nursing program providers did not offer MHFA training, seven of the eleven program representatives (N3, N4, N5, N9, N10, N12, N13) interviewed reported encouraging students to seek MHFA training offered by their universities. Four of eleven nursing program providers (N4, N6, N12, N13) cited insufficient time in the academic calendar as a barrier to implementation: *“they were considering it, but there was too much else put in the curriculum,”* (N4). All eleven program providers specified that they provided mental health units required for curricula accreditation, of which ten program providers reported that they (N1, N3, N4, N5, N6, N8, N9, N10, N12, N13) had mental health units incorporating content related to MHFA, including skills relating to suicide care. One nursing program provider (N7) stated that their mental health units did not teach any MHFA skills. The N1 and N5 representatives felt that mental health content within their core curricula exceeded the skills gained through MHFA training, as explained by N5: “*our expectation is that the content that we deliver … is equal to and greater than that in the MHFA course*”. One program provider (N3) intended to pilot MHFA in 2020 but was unable to due to COVID-19’s effects on tertiary teaching. Two (N7, N8) of the 11 nursing program providers had previously provided MHFA training but discontinued it due to insufficient funding despite reporting that MHFA added value or improved comfortability among students and expanded their clinical skillset. N8 explained, “*I think it (MHFA) should be done more widely, especially for those that don’t do any mental health courses. The nurses do mental health courses, but for the likes of pharmacists and everyone who comes into contact with the general public… it’s a really good thing for them to have a background in it”*.

Of the four pharmacy program providers (P4, P5, P6, P10) that did not provide MHFA training, two (P5, P10) representatives indicated intentions of offering MHFA in 2020 or 2021. Program provider P5 had previously offered MHFA training but could no longer offer the training due to lack of staffing. Instead, P5 intended to embed compulsory MHFA training by engaging with external instructors. P6 had no plans to implement MHFA admitting, ***“****it comes [down] to the cost perspective… we do things like vaccinations but it [MHFA] hasn’t been one of those things that have been completely on our radar to subsidise”* (P6).

## Discussion

As the first national study investigating MHFA training and assessment across accredited Australian medicine, nursing and pharmacy program providers, this study captured data on MHFA training and assessment among 59, 35 and 56% of all medicine, nursing and pharmacy program providers, nationally. Of those interviewed (*n* = 36), 42% of program providers were offering MHFA within their program(s). In addition to barriers and facilitators influencing uptake, such as time, perceptions of relevance to professional practice and timetabling, this study uncovered the lack of opportunities offered for students to practice MHFA skills through activities and assessments post-training, which may impact students’ confidence in applying MHFA in practice.

Funding granted to MDANZ increased the accessibility of MHFA among all medical program providers. However, this funding only provides access to the eLearning component of MHFA. Without completing the instructor-led component and associated assessment, students are not able to qualify for MHFA accreditation to become MHFAiders [22]. Among UK medical students, the eLearning course was found to improve knowledge, intended actions and confidence, whilst reducing stigma in relation to people experiencing mental health problems [36]. Tailored MHFA eLearning for medical and nursing students was found to be similarly effective to the standard course; however, randomised controlled trials comparing outcomes among healthcare students are lacking [16]. Similarly, a study comparing Blended MHFA training with the eLearning course among public servants found comparable positive effects on knowledge, treatment beliefs, intentions and confidence to help; however, participants who completed Blended MHFA were significantly more likely to report that it was more useful, that they learnt “a great deal” and that they would recommend the course to others [37]. Despite promising preliminary evidence demonstrating positive outcomes from Blended and eLearning MHFA courses, participants seem to prefer completing a MHFA course involving an instructor-led component, warranting further investigation. Furthermore, studies comparing outcomes among healthcare students to explore the sustainability of impact are also needed. With funding granted for only 2 years [22], ongoing MHFA training by medical program providers is uncertain as some programs may cease offering the training without funding.

Nursing program providers’ low rate of MHFA delivery was often attributed to the perception that mental health content required for program accreditation would cover or exceed skills taught in MHFA training, required for clinical practice. Existing research supporting this perception has outlined that nursing curricula increases student’s mental health knowledge [38] with clinical placements significantly improving attitudes and confidence in caring for people experiencing mental illnesses [39]. There is also concern that the inclusion of MHFA training into core curricula would result in the exclusion of other crucial mental health nursing [40]. A recent study assessing mental health literacy levels in Australian nursing students revealed that 40% of students reported not having sufficient mental health literacy for practice [41]. There is endorsement for MHFA training to become a pre-requisite for all prospective nursing students, thereby providing a foundation for more advanced mental health content without burdening an already overcrowded curriculum [40, 41]. Although MHFA should not replace existing mental health content in nursing curricula, the introduction of MHFA in the early stages of the nursing program may improve desirability of mental health nursing as a career choice [42].

There was a high rate of MHFA training delivery among pharmacy program providers, particularly for final-year students. Placement of training in the final year enables graduates to enter the workforce with valid accreditation [15]. Furthermore, two of the three program providers that incorporated post-training assessment in curricula were pharmacy program providers. International literature also reflects that pharmacy program providers most commonly offer MHFA training and assessment to university students [15]. Some of the earliest studies exploring MHFA training among university students were among pharmacy students, indicating that the training reduces stigma whilst improving confidence and literacy [18]. The availability of evidence supporting effectiveness among pharmacy students may have contributed to higher levels of uptake among pharmacy program providers. Furthermore, mental health education is an area of need for the pharmacy profession [43, 44], potentially further contributing to pharmacy program providers’ willingness to provide MHFA training.

An apparent lack of activities allowing students to practice and be assessed on MHFA skills across interviewed program providers means that students are not given opportunities to adequately consolidate newly-acquired skills. This is consistent with preliminary evidence among pharmacy students suggesting that MHFA participants may over- and under-estimate their ability to provide MHFA when compared to observed performance during simulated assessments [45]. Similarly, among primary education degree students, asthma first aid training led to significant improvements in knowledge, assessed by a questionnaire; however, only 29% could demonstrate a level of competency required to assist a child experiencing severe asthma exacerbation [46]. Hence, the addition of training components that require demonstration of competency have been recommended [47]. While the evidence relating to competence post-MHFA training among students, specifically, is lacking [15]; this phenomenon appears to be common in the literature among students and healthcare professionals, more generally, and is a topic worthy of investigation. For example, therapists’ self-reported identification as a cognitive behavioural therapy (CBT) therapist did not accurately reflect actual provision of CBT services during sessions as rated by expert observers [48]. Therefore, further opportunities for participants to demonstrate skills post-training, such as through objective assessment methods, are recommended. Studies exploring students’ perceived confidence and ability to apply MHFA in simulated and real-world settings long-term are needed to determine the true value of MHFA to recipients beyond its demonstrated effectiveness in improving self-reported constructs [49].

With national suicide-related deaths increasing [50], inconsistencies in MHFA training provision among program providers and the clear benefit of MHFA training [14], the question arises as to whether MHFA training should become a pre-requisite for registration as a healthcare professional. International healthcare systems, including The Washington State Department of Health, have made suicide prevention courses compulsory for healthcare workers having recognised their role in supporting approximately 40% of people with suicidal ideation who visit healthcare infrastructures within a week of attempting suicide [51]. Currently in Australia, only physical first aid and cardiopulmonary resuscitation training is a mandatory minimum standard for the accreditation of doctors [52], and pharmacists [53] and is a work health and safety requirement for all workplaces [54]. In contrast, there is no mandatory minimum standard for mental health education in the Australian healthcare or general workforce. Endorsement by pharmacy student organisations [55] and funding by the Australian Government [21, 22] demonstrates widespread support for MHFA training for healthcare professionals. Internationally, there has also been support by other organisations, such as the American Pharmacists’ Association [56]. If MHFA were to become a national minimum standard for mental health and crisis first aid education in the Australian healthcare workforce, funding needs to be considered. For example, the current reliance on limited government funding for training provision by many medical program providers is likely to lead to uncertainty in the future of MHFA training. Without further and continued funding from government bodies, it may become the responsibility of the individual healthcare worker to seek MHFA training.

### Strengths and limitations

This study is the first to explore and identify the extent of MHFA training delivery and assessment across Australian medical, nursing and pharmacy programs, yet it is important to consider the findings in the context of potential study limitations. This study explored MHFA training and assessment among medicine, nursing and pharmacy program providers because doctors, nurses and pharmacists collectively represent over 75% of the health workforce [20]. Approximately 47% of all national accredited program providers were interviewed, identifying that 42% of those interviewed offer MHFA training. However, the rate of MHFA training may be different among program providers that did not participate in this study, presenting a potential source of selection bias. Nonetheless, program providers interviewed were geographically distributed across all Australian states and territories except for NT and 47% of accredited providers nationally were interviewed, demonstrating the representativeness of the sample interviewed in this study. Only the NT was not represented in this study; however, with their only pharmacy program provider no longer taking enrolments from 2020 [57], no medical program providers and one nursing program provider, the lack of data captured from the NT is unlikely to have impacted the results, lending evidence to the generalisability of the findings. Furthermore, representatives from 21 program providers who did not provide MHFA at the time of interview, participated in this study which also reduces the potential for bias. Another strength of this study is the spread of representatives interviewed among each of the disciplines, with 59% (*n* = 13) of the total medical, 35% (*n* = 13) of the total nursing and 56% (*n* = 10) of the total pharmacy program providers being interviewed and an overall participation rate of 47%.

Another potential limitation is that one co-author of this study was interviewed as a program provider representative due to their role in MHFA organisation and training within their program. Furthermore, it should be noted that the accuracy of the data collected was reliant upon an interviewee’s memory and their understanding of MHFA training and accreditation. Some had difficulty recalling information and were only able to give estimates, especially when interviewees’ employment had commenced after MHFA was introduced. These estimates were reported as specified by the interviewees. No assumptions were made by the authors; however, the findings must be interpreted with the knowledge that they are based on the interviewees’ recall and understanding of MHFA training.

## Conclusion

MHFA training is increasingly recognised as integral for future healthcare professionals, with 42% of accredited medical, nursing and pharmacy program providers interviewed offering MHFA training to students. Inconsistencies in MHFA training delivery and assessment were apparent, and there is a lack of consensus as to which student cohorts should receive training, which type of MHFA training is most appropriate, and whether post-training activities and assessments are needed. Although MHFA training has proven instrumental in increasing mental health knowledge, attitudes and confidence among healthcare students, barriers to MHFA implementation included available funding, staffing and scheduling. Addressing barriers would potentially allow for MHFA training to become a minimum standard of mental health and crisis first aid education for doctors, nurses and pharmacists. Further research exploring application of MHFA through post-training assessments and in real-world settings, as well as MHFA implementation in other healthcare degree programs is needed.

## Data Availability

Not applicable.

## Abbreviations

MHFA: Mental Health First Aid
MDANZ: Medical Deans Australia and New Zealand
AHPRA: Australian Health Practitioner Regulation Agency
FTF: Face-to-face
MHFAiders: Mental Health First Aiders
NT: Northern Territory
NSW: New South Wales
QLD: Queensland
WA: Western Australia
VIC: Victoria
SA: South Australia
TAS: Tasmania

## References

[1] World Health Organization (2021). Suicide.

[2] Sher L (2011). Teaching medical professionals about suicide prevention: what’s missing?. QJM: Int J Med.

[3] Mental Health First Aid Australia. What we do at mental health first aid: Mental Health First Aid Australia; 2021. [cited 2021 Jan 20]. Available from: https://mhfa.com.au/about/our-activities/what-we-do-mental-health-first-aid

[4] Mental Health First Aid Australia (2021). Welcome to mental health first aid Australia.

[5] Kitchener BA, Jorm AF, Kelly CM. Mental health first aid manual. 4th ed. Melbourne: Mental Health First Aid Australia; 2017.

[6] Kitchener BA, Jorm AF (2017). The role of mental health first aid training in nursing education: A response to Happell, Wilson & McNamara (2015). Collegian..

[7] Jorm AF, Kitchener BA (2011). Noting a landmark achievement: mental health first aid training reaches 1% of Australian adults. Austral New Zealand J Psychiatry.

[8] Boukouvalas E, El-Den S, Murphy AL, Salvador-Carulla L, O'Reilly CL (2020). Exploring health care professionals’ knowledge of, attitudes towards, and confidence in caring for people at risk of suicide: a systematic review. Arch Suicide Res.

[9] Scharwtz S (2019). Educating the nurse of the future - report of the independent review of nursing education Canberra: commonwealth of Australia.

[10] Thomas S, Pai N, Dawes K, Wilson C, Williams V. Updating medical school psychiatry curricula to meet projected mental health needs. Australasian Psychiatry. 2013;21(6):578-82. 10.1177/1039856213500092 23996665

[11] Mey A, Hattingh HL, Davey A, Knox K, Fejzic J, Wheeler A (2015). Preparing community pharmacists for a role in mental health: an evaluation of accredited Australian pharmacy programs. Curtin’s Inst Reposit.

[12] Cowlishaw S, Metcalf O, Varker T, Stone C, Molyneaux R, Gibbs L, et al. Anger Dimensions and Mental Health Following a Disaster: Distribution and Implications After a Major Bushfire. Journal of Traumatic Stress. 2021;34(1):46-55. 10.1002/jts.22616 33136348

[13] Moreno C, Wykes T, Galderisi S, Nordentoft M, Crossley N, Jones N (2020). How mental health care should change as a consequence of the COVID-19 pandemic. Lancet Psychiatry.

[14] Morgan AJ, Ross A, Reavley NJ (2018). Systematic review and meta-analysis of mental health first aid training: effects on knowledge, stigma, and helping behaviour. PLoS One.

[15] El-Den S, Moles R, Choong HJ, O'Reilly C (2020). Mental health first aid training and assessment among university students: a systematic review. J Am Pharm Assoc (2003).

[16] Bond KS, Jorm AF, Kitchener BA, Reavley NJ (2015). Mental health first aid training for Australian medical and nursing students: an evaluation study. BMC Psychol.

[17] Burns S, Crawford G, Hallett J, Hunt K, Chih HJ, Tilley PJ (2017). What's wrong with John? A randomised controlled trial of mental health first aid (MHFA) training with nursing students. BMC Psychiatry.

[18] O'Reilly CL, Bell JS, Kelly PJ, Chen TF (2011). Impact of mental health first aid training on pharmacy students’ knowledge, attitudes and self-reported behaviour: a controlled trial. Aust N Z J Psychiatry.

[19] Dutheil F, Aubert C, Pereira B, Dambrun M, Moustafa F, Mermillod M (2019). Suicide among physicians and health-care workers: A systematic review and meta-analysis. PLoS One.

[20] Commonwealth of Australia (2020). Health workforce summaries.

[21] Mental Health First Aid Australia (2021). Tertiary students.

[22] Medical Deans Australia and New Zealand (2020). Medical students get support to train in mental health first aid.

[23] Pharmaceutical Society of Australia (2020). Mental health first aid - bushfire affected Australians explained.

[24] The Pharmacy Guild of Australia (2021). Mental health community pharmacy program 2020: the Pharmacy Guild of Australia.

[25] Haggan M. Pharmacists ‘a wonderful and valued resource’ in mental health. Austral J Pharm. 2020; [cited 2020 Dec 21]. Available from: https://ajp.com.au/news/pharmacists-a-wonderful-and-valued-resource-in-mental-health/.

[26] Commonwealth of Australia (2020). Mental health support available for rural frontline nurses.

[27] Byrne K, McGowan I, Cousins W (2015). Delivering mental health first aid: an exploration of instructors’ views. Int J Ment Health Promot.

[28] Mental Health First Aid Australia (2021). MHFA skilled workplace program.

[29] Terry J (2011). Delivering a basic mental health training programme: views and experiences of mental health first aid instructors in Wales. J Psychiatr Ment Health Nurs.

[30] Kitchener BA, Jorm AF (2008). Mental health first aid: an international programme for early intervention. Early Interv Psychiatry.

[31] Australian Journal of Pharmacy (2016). Pharmacy's role in mental health AUS.

[32] AHPRA & National Boards (2020). Approved programs of study Melbourne.

[33] Crowe M, Inder M, Porter R (2015). Conducting qualitative research in mental health: thematic and content analyses. Aust N Z J Psychiatry.

[34] Mental Health First Aid Australia (2021). Standard mental health first aid.

[35] Mental Health First Aid Australia (2021). Our Impact.

[36] Davies EB, Beever E, Glazebrook C (2018). A pilot randomised controlled study of the mental health first aid eLearning course with UK medical students. BMC Med Educ.

[37] Reavley NJ, Morgan AJ, Fischer JA, Kitchener B, Bovopoulos N, Jorm AF (2018). Effectiveness of eLearning and blended modes of delivery of mental health first aid training in the workplace: randomised controlled trial. BMC Psychiatry.

[38] Neville C, Goetz S (2014). Quality and substance of educational strategies for mental health in undergraduate nursing curricula. Int J Ment Health Nurs.

[39] Foster K, Withers E, Blanco T, Lupson C, Steele M, Giandinoto JA (2019). Undergraduate nursing students’ stigma and recovery attitudes during mental health clinical placement: A pre/post-test survey study. Int J Ment Health Nurs.

[40] Happell B, Wilson R, McNamara P (2015). Undergraduate mental health nursing education in Australia: more than mental health first aid. Collegian..

[41] Saito AS, Creedy DK (2021). Determining mental health literacy of undergraduate nursing students to inform learning and teaching strategies. Int J Ment Health Nurs.

[42] Crawford G, Burns S (2020). Confidence and motivation to help those with a mental health problem: experiences from a study of nursing students completing mental health first aid (MHFA) training. BMC Med Educ.

[43] Wheeler A, Mey A, Kelly F, Hattingh L, K. Davey A. (2014). Education and training for community pharmacists in mental health practice: how to equip this workforce for the future. J Ment Health Train Educ Pract.

[44] Kirschbaum M, Peterson G, Bridgman H (2016). Mental health first aid training needs of Australian community pharmacists. Curr Pharm Teach Learn.

[45] El-Den S, Chen TF, Moles RJ, O'Reilly C (2018). Assessing mental health first aid skills using simulated patients. Am J Pharm Educ.

[46] Luckie K, Saini B, Galstaun V, Kritikos V, Collins JC, Moles RJ (2018). The effectiveness of an online training programme to prepare teachers to provide asthma first aid. J Paediatr Child Health.

[47] Luckie K, Saini B, Soo YY, Kritikos V, Collins JC, Moles RJ (2019). Impact of scenario based training on asthma first aid knowledge and skills in school staff: an open label, three-arm, parallel-group repeated measures study. J Asthma.

[48] Creed TA, Wolk CB, Feinberg B, Evans AC, Beck AT (2016). Beyond the label: relationship between community therapists’ self-report of a cognitive behavioral therapy orientation and observed skills. Adm Policy Ment Health Ment Health Serv Res.

[49] Mei C, McGorry PD (2020). Mental health first aid: strengthening its impact for aid recipients. Evid Based Ment Health.

[50] Australian Institute of Health and Welfare (2020). Deaths by suicide over time.

[51] Washington State Department of Health (2020). Suicide prevention training for health professions.

[52] The Royal Australian College of General Practitioners (2021). Basic life support.

[53] Pharmacy Board of Australia (2021). Frequently asked questions- continuing professional development for pharmacists and pharmacy interns.

[54] Safe Work Australia (2012). First aid in the workplace code of practice.

[55] National Australian Pharmacy Students’ Association (2016). Position statement: mental health first aid education.

[56] McKee J, Mospan CM, Benfield M, Gillette C (2003). A call for community pharmacists to complete mental health first aid training. J Am Pharm Assoc.

[57] Haggan M. NT pharmacy faces uncertain future [internet]. Austral J Pharm. 2019; [cited 2021 Jan 31]. Available from: https://ajp.com.au/news/nt-faces-uncertain-future/.

